# Physiological and Transcriptomic Analyses of the Thermophilic, Aceticlastic Methanogen *Methanosaeta thermophila* Responding to Ammonia Stress

**DOI:** 10.1264/jsme2.ME14021

**Published:** 2014-06-10

**Authors:** Souichiro Kato, Konomi Sasaki, Kazuya Watanabe, Isao Yumoto, Yoichi Kamagata

**Affiliations:** 1 Bioproduction Research Institute, National Institute of Advanced Industrial Science and Technology (AIST), 2–17–2–1 Tsukisamu-Higashi, Toyohira-ku, Sapporo, Hokkaido 062–8517, Japan; 2 Division of Applied Bioscience, Graduate School of Agriculture, Hokkaido University, Kita-9 Nishi-9, Kita-ku, Sapporo, Hokkaido 060–8589, Japan; 3 Research Center for Advanced Science and Technology, The University of Tokyo, 4–6–1 Komaba, Meguro-ku, Tokyo 153–8904, Japan; 4 Hokkaido High-Technology College, 2–12–1 Megumino-kita, Eniwa, Hokkaido 061–1374, Japan; 5 School of Life Sciences, Tokyo University of Pharmacy and Life Sciences, 1432–1 Horinouchi, Hachioji, Tokyo 192–0392, Japan

**Keywords:** ammonia stress, methanogenesis, *Methanosaeta thermophila*, syntrophic acetate oxidation, transcriptome

## Abstract

The inhibitory effects of ammonia on two different degradation pathways of methanogenic acetate were evaluated using a pure culture (*Methanosaeta thermophila* strain PT) and defined co-culture (*Methanothermobacter thermautotrophicus* strain TM and *Thermacetogenium phaeum* strain PB), which represented aceticlastic and syntrophic methanogenesis, respectively. Growth experiments with high concentrations of ammonia clearly demonstrated that sensitivity to ammonia stress was markedly higher in *M. thermophila* PT than in the syntrophic co-culture. *M. thermophila* PT also exhibited higher sensitivity to high pH stress, which indicated that an inability to maintain pH homeostasis is an underlying cause of ammonia inhibition. Methanogenesis was inhibited in the resting cells of *M. thermophila* PT with moderate concentrations of ammonia, suggesting that the inhibition of enzymes involved in methanogenesis may be one of the major factors responsible for ammonia toxicity. Transcriptomic analysis revealed a broad range of disturbances in *M. thermophila* PT cells under ammonia stress conditions, including protein denaturation, oxidative stress, and intracellular cation imbalances. The results of the present study clearly demonstrated that syntrophic acetate degradation dominated over aceticlastic methanogenesis under ammonia stress conditions, which is consistent with the findings of previous studies on complex microbial community systems. Our results also imply that the co-existence of multiple metabolic pathways and their different sensitivities to stress factors confer resiliency on methanogenic processes.

Anaerobic methanogenic digestion is widely used for the treatment of diverse organic waste and wastewater. Methanogenic digesters operated under thermophilic conditions (approximately 55°C) have attracted considerable attention due to their numerous advantages over mesophilic digesters, including the potential for higher organic decomposition and methane production, pathogen reduction, and reduced foaming (35). In spite of these benefits, thermophilic methanogenic digestion is susceptible to a number of environmental factors, such as temperature, pH, and inhibitory substances (5, 6). One of the most harmful stressors that affects thermophilic digesters is high ammonia concentrations (NH_4_^+^ and NH_3_), which are produced primarily as a consequence of the degradation of nitrogenous compounds including proteins and urea (5, 18, 34). The inhibition of methanogenesis is frequently observed in methanogenic digestion processes that treat waste containing high levels of nitrogenous compounds, such as livestock manure and dewatered sewage sludge (1, 2, 8, 36). In aqueous solution, inorganic ammonia nitrogen exists as either free ammonia (NH_3_) or ammonium ion (NH_4_^+^) in equilibrium. Free ammonia is the main inhibitor of methanogenesis due to its higher membrane permeability (33). A previous study demonstrated that thermophilic methanogenic digesters became more susceptible to the accumulation of ammonia than mesophilic digesters when the ratio between free ammonia and ammonium ions increased with increasing temperatures (3, 4).

Ammonia inhibition in methanogenic digesters typically results in a decrease in the production of methane and concomitant accumulation of volatile fatty acids, particularly acetate, which suggests that methanogenic acetate degradation is susceptible to ammonia stress (3, 8, 23, 27, 39). Methanogenic acetate degradation proceeds by either aceticlastic methanogenesis or syntrophic acetate oxidation. In the aceticlastic pathway, the methyl group of acetate is converted to methane and the carboxyl group is oxidized to CO_2_ (12). This form of methanogenesis is mediated by aceticlastic methanogens represented by *Methanosaeta* spp. and *Methanosarcina* spp. In contrast, in the syntrophic acetate oxidation pathway, acetate is first oxidized to H_2_ and CO_2_ by syntrophic acetate-oxidizing bacteria (SAOB), followed by hydrogenotrophic methanogenesis (42). The acetate oxidation reaction is endergonic under standard conditions and is only feasible under a low H_2_ partial pressure; therefore, the H_2_ scavenging reaction by hydrogenotrophic methanogens is essential to make the oxidation of acetate by SAOB energetically favorable (10, 14). Syntrophic acetate oxidation is a relatively slow reaction because of thermodynamic constraints. Hence, aceticlastic methanogenesis is the primary methanogenic acetate degradation pathway in thermophilic methanogenic digesters operated under optimum conditions. However, microbial community and methanogenic pathway analyses in diverse methanogenic digesters and enrichment cultures revealed that syntrophic acetate degradation dominated over aceticlastic methanogenesis under ammonia stress conditions (14, 19, 24, 28, 29, 38), while the opposite has also been reported (7, 41). These findings have been explained by differences in the sensitivity to ammonia; the sensitivity of aceticlastic methanogens to ammonia appears to be markedly higher than that of SAOB and hydrogenotrophic methanogens. However, these studies were based solely on community analysis, and no explicit comparative investigation has been conducted on the inhibition of ammonia using pure cultures of aceticlastic methanogens and defined co-cultures of SAOB and hydrogenotrophic methanogens. Furthermore, even though several mechanisms have been proposed, including elevations in intracellular pH, disturbances in intracellular cation levels, and the inhibition of specific enzymatic reactions, those responsible for the ammonia inhibition of methanogenic microorganisms remain unclear (15, 33, 40).

In the present study, the inhibitory effects of ammonia were quantitatively investigated based on a representative pure and defined two-membered co-culture represented by thermophilic aceticlastic methanogens (*Methanosaeta thermophila* strain PT), and SAOB (*Thermacetogenium phaeum* strain PB) with hydrogenotrophic methanogens (*Methanothermobacter thermautotrophicus* strain TM).

## Materials and Methods

### Archaeal and bacterial strains and culture conditions

*M. thermophila* PT (DSM 6194^T^) (13) and *T. phaeum* PB (DSM 12270^T^) (9) were obtained from the Deutsche Sammlung von Mikroorganismen und Zellkukturen GmbH (Braunschweig, Germany). *M. thermautotrophicus* TM was isolated from a thermophilic anaerobic methanogenic reactor in Japan (9). Unless otherwise stated, cultivations were conducted at 55°C with 68-mL capacity serum vials containing 20 mL of a bicarbonate-buffered inorganic medium (pH 7.0) under an atmosphere of N_2_-CO_2_ (4:1 [v/v]) without shaking. The inorganic medium contained the following chemicals (per liter): 0.3 g of KH_2_PO_4_, 0.27 g of NH_4_Cl, 0.1 g of MgCl_2_·7H_2_O, 0.08 g of CaCl_2_·7H_2_O, 0.6 g of NaCl, 3.5 g of KHCO_3_, and 10 mL each of the trace metal solution and vitamin solution (31). The reducing agents (0.3 g L^−1^ each of Na_2_S·9H_2_O and cysteine-HCl·H_2_O) were supplemented to the medium from filter-sterilized stock solutions after autoclaving. Methanol (40 mM) or 200 kPa H_2_-CO_2_ (4:1 [v/v]) were supplemented as energy and carbon sources for the pure cultures of *T. phaeum* PB and *M. thermautotrophicus* TM, respectively. Sodium acetate (40 mM) was utilized as an energy and carbon source for the pure culture of *M. thermophila* PT, the defined co-culture of *T. phaeum* PB and *M. thermautotrophicus* TM, and the tri-culture of the three strains. In the ammonia and NaCl stress experiments, the sterilized culture medium was supplemented with anaerobic, filter-sterilized, 5 M stock solutions of NH_4_Cl or NaCl to give desired concentrations. In the high-pH stress experiments, the pH of the medium was adjusted to appropriate values with 1 N HCl or 1 N NaOH after sterilization. The medium pH was not influenced by the NH_4_Cl amendment (up to 500 mM) and was kept constant during the incubation (initial pH values ± 0.2). In the experiments with resting cells, the gas phase of the early stationary phase cultures (day 7) of *M. thermophila* PT grown on 40 mM acetate was exchanged with N_2_/CO_2_ (4:1 [v/v]) gas and the cultures were supplemented with 40 mM acetate and NH_4_Cl, followed by an incubation at 55°C. The growth of *T. phaeum* PB was monitored by measuring the optical density at 600 nm. The production of methane in *M. thermophila* PT and *M. thermautotrophicus* TM in pure and mixed cultures was analyzed using a gas chromatograph (GC-2014, Shimadzu) equipped with a thermal conductivity detector, flame ionization detector, and molecular sieve 13X column (Shimadzu). The column, injection, and detector temperatures were 70, 180, and 200°C, respectively. The methane production rates were calculated as described elsewhere (26) and used as the indicator of their growth rate.

### RNA isolation

Total RNA was isolated using ISOGEN II reagent (Nippon Gene, Japan) combined with a bead-beating method. The cell pellet was suspended in 1 mL ISOGEN II reagent and then transferred to 2-mL polypropylene tubes containing silica beads (Lysing Matrix E, MP Biomedicals). Two-hundred microliters of chloroform was added to the tubes, and the resulting slurry was homogenized in a Fastprep 24 instrument (version 4, MP Biomedicals) for 60 s at a speed of 6.0. The tube was centrifuged (15,000×*g*, 4°C, 5 min) and the aqueous layer was recovered. Total RNA was purified using an RNeasy Mini kit (Qiagen) with DNase treatment (RNase-free DNase set, Qiagen) as described in the manufacturer’s instructions. The purified RNA was spectroscopically quantified using a NanoDrop ND-1000 spectrophotometer (NanoDrop Technologies).

### Transcriptome analysis on *M. thermophila* PT

Regarding the transcriptome analysis, *M. thermophila* PT (20 mL of pre-cultivated cells) was inoculated into 400 mL of the inorganic medium supplemented with 40 mM sodium acetate in a 1.3-L capacity serum bottle and was cultivated until its exponentially growing phase (day 4), followed by supplementation with NH_4_Cl at a final concentration of 100 mM. Cells for the RNA isolation were collected after a further incubation for 1 h. Specific oligonucleotides (60-mers) were designed for 1,671 genes (corresponding to 98.5% of the total predicted protein-coding sequences in the *M. thermophila* PT genome) (32) using the eArray protocol and fabricated on glass slides by SurePrint technology (Agilent Technologies). Six spots, consisting of 6 different sequences, were printed on the array for each gene. The mean value of the normalized signal intensities of the 6 spots was used for the analysis. The fluorescence labeling of cDNA, hybridization, and scanning of hybridized arrays were conducted as previously described for the two-color microarray-based gene-expression analysis (15–17). To minimize dye biases, dyes (Cy3 and Cy5) were swapped for each replicate. Total RNA isolated from a *M. thermophila* PT culture without ammonia supplementation was used as the reference sample. Fluorescence-dye labeled cDNA for the ammonia-treated culture was mixed with oppositely labeled cDNA for the reference sample and hybridized with the probes on the microarray. RNA samples from four cultures separately cultivated under the same conditions were analyzed as biological replicates. Data acquisition, normalization, and statistical analysis were performed as described previously (15–17) using Feature Extraction Software version 8.1 (Agilent Technologies) and GeneSpring GX ver. 10 (Agilent Technologies). Microarray data were deposited in the ArrayExpress database under the accession number E-MEXP-3849.

### Quantitative reverse transcription-PCR (qRT-PCR)

The PCR primers used for qRT-PCR were designed with Primer3 software (http://simgene.com/Primer3) and are listed in Table S1. The quantification of 16S rRNA copy numbers in the defined mixed culture and quantitative gene expression analysis of *M. thermophila* PT were performed by one-step real-time RT-PCR using a Mx3000P QPCR System (Stratagene) and RNA-direct SYBR Green Realtime PCR Master Mix (Toyobo) according to the manufacturer’s instructions. Real-time RT-PCR was started with reverse transcription at 61°C for 20 min. After an initial denaturation at 95°C for 1 min, target cDNA was amplified by 40 cycles of denaturation for 15 s at 95°C, followed by annealing and extension for 45 s at 60°C. Fluorescence was detected at the end of each extension reaction. The purity of PCR products was checked by melting curve analysis. At least three biological replicates were subjected to qRT-PCR analysis, and at least two separate trials were conducted for each sample. Standard curves were generated with serially diluted PCR products (10^3^–10^8^ copies mL^−1^, calculated from the quantity of DNA and the expected length of the PCR products) amplified using the respective primer sets and were used to calculate the copy number of rRNA or mRNA in the total RNA samples. In the quantitative gene expression analysis, the copy numbers of rRNA in each sample were used for normalization among the samples.

## Results and Discussion

### Effects of ammonia on the representative microorganisms of aceticlastic and syntrophic methanogenesis

**Fig. 1:**
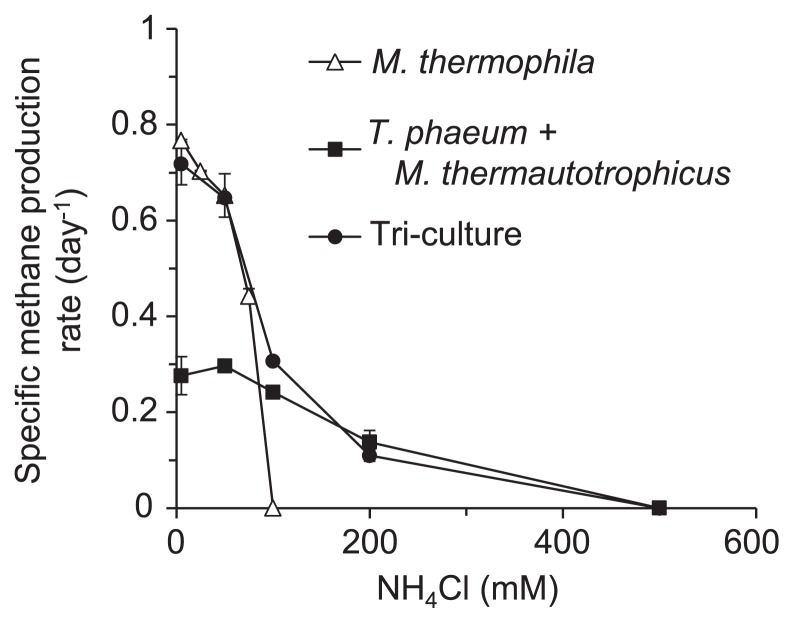
Effects of ammonia on aceticlastic and syntrophic methanogenesis from acetate. The methane production rates of a pure culture of *M. thermophila* PT (open triangles), co-culture of *T. phaeum* PB and *M. thermautotrophicus* TM (filled squares), and tri-culture of the three strains (filled circles) are plotted against NH_4_Cl concentrations. Data are presented as the means of three independent cultures, and error bars represent standard deviations.

The thermophiles used in this study (*M. thermophila* PT, *M. thermautotrophicus* TM, and *T. phaeum* PB) were originally isolated from a thermophilic methanogenic digester (9, 13), and are considered to be representative species for the methanogenic acetate degradation reactions that occur in diverse thermophilic methanogenic digesters (11, 22, 30) and various natural environments, such as high-temperature petroleum reservoirs (20, 21, 25). The pure culture of *M. thermophila* PT and defined co-culture of *T. phaeum* PB and *M. thermautotrophicus* TM were cultivated with different concentrations of NH_4_Cl and their methane production rates were then determined as an indicator of their growth rates (Fig. 1). The susceptibility of *M. thermophila* PT to ammonia stress was higher than that of the syntrophic co-culture. At 75 mM NH_4_Cl, the methane production rate of *M. thermophila* PT was reduced by approximately 50%, and no methanogenesis was observed in cultures supplemented with ≥100 mM NH_4_Cl. In contrast, the methane production rate of the syntrophic co-culture was barely affected by 100 mM NH_4_Cl, but was reduced by nearly 50% with 200 mM NH_4_Cl. The inhibitory effects of ammonia on the pure cultures of *T. phaeum* PB and *M. thermautotrophicus* TM were also assessed (Fig. S1), suggesting that these microbial species may be significantly more tolerant than *M. thermophila* PT.

### Effects of ammonia on the defined tri-culture of *M. thermophila* PT, *M. thermautotrophicus* TM, and *T. phaeum* PB

The inhibitory effects of ammonia on the methane production rates of a defined tri-culture of *M. thermophila* PT, *M. thermautotrophicus* TM, and *T. phaeum* PB were also assessed (Fig. 1). In the tri-culture, the two main pathways for acetate utilization, namely aceticlastic methanogenesis by *M. thermophila* PT and syntrophic acetate degradation by *T. phaeum* PB/*M. thermautotrophicus* TM, are in direct competition, reflecting the environment in methanogenic digesters. The methane production rates of the tri-culture supplemented with 5 or 50 mM NH_4_Cl were similar to those of *M. thermophila* PT in pure culture, while the methane production rates of the tri-culture supplemented with 100 or 200 mM NH_4_Cl were similar to those of the syntrophic co-culture (Fig. 1). These results suggested that the methanogenic pathway may have transitioned from aceticlastic to syntrophic acetate degradation at NH_4_Cl concentrations between 50 and 100 mM.

**Fig. 2:**
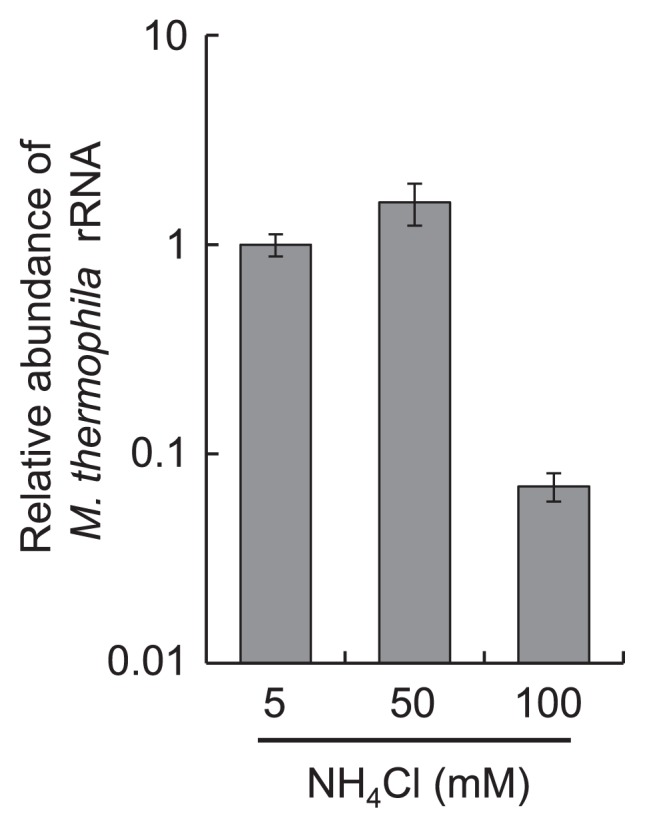
Effects of ammonia on the growth of *M. thermophila* PT in the tri-culture. Abundances of rRNA of *M. thermophila* PT in the mid-log phase of the tri-culture grown on acetate with different concentrations of NH_4_Cl were determined by qRT-PCR and normalized against that of the control cultures (5 mM NH_4_Cl). Data are presented as the means of three independent cultures, and error bars represent standard deviations.

To confirm the transition of the methanogenic pathway, the abundance of *M. thermophila* PT at the mid-log phase of the tri-culture was determined by qRT-PCR targeting 16S rRNA. The relative abundance of *M. thermophila* PT was not affected by the addition of 50 mM NH_4_Cl to the culture, whereas 100 mM NH_4_Cl markedly decreased the abundance of *M. thermophila* PT to less than 10% of that found in the control cultures (5 mM NH_4_Cl) (Fig. 2).

These results clearly demonstrated that the aceticlastic methanogen *M. thermophila* PT was less tolerant of ammonia stress than the two other examined species; hence, syntrophic acetate degradation outcompeted aceticlastic methanogenesis under high ammonia concentrations. These results are consistent with the previous findings of microbial community and methanogenic pathway analyses using methanogenic digesters (14, 19, 24, 28, 29, 38).

### Effects of NaCl on the aceticlastic and syntrophic cultures

**Fig. 3:**
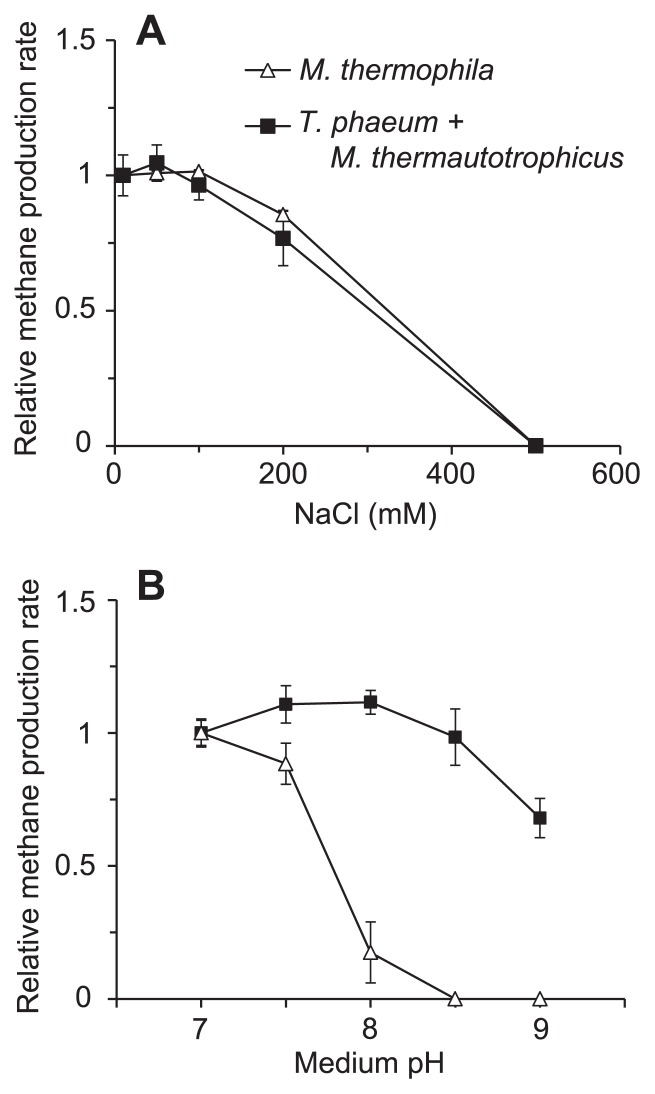
Effects of NaCl and pH on aceticlastic and syntrophic methanogenesis from acetate. The methane production rates of a pure culture of *M. thermophila* PT (open triangles) and co-culture of *T. phaeum* PB and *M. thermautotrophicus* TM (filled squares) are plotted against NaCl concentrations (A) and medium pH values (B). Methane production rates were normalized against those of the control cultures (10.3 mM NaCl, pH 7.0, and 5 mM NH_4_Cl). Data are presented as the means of three independent cultures, and error bars represent standard deviations.

To determine whether increases in medium osmolality and/or ionic strength were causal factors of ammonia inhibition, the pure culture of *M. thermophila* PT and syntrophic co-culture were cultivated in medium supplemented with various concentrations of NaCl. The methane production rates of these cultures showed similar patterns in response to NaCl concentrations (Fig. 3A). The methane production rate was not affected at NaCl concentrations of ≤100 mM and was only partly inhibited (20–30% decrease in the methane production rate) by 200 mM NaCl. Since 100 mM NH_4_Cl completely inhibited the methanogenesis of *M. thermophila* PT (Fig. 1), it was concluded that increases in osmolality and/or ionic strength were not the primary factors underlying ammonia inhibition of *M. thermophila* PT.

### Effects of alkaline pH on the aceticlastic and syntrophic cultures

The perturbation of pH homeostasis by elevated pH has been speculated to be a possible cause of ammonia inhibition (33, 40). To evaluate the inhibitory effects of pH homeostatic disruption on growth and methanogenesis, the pure culture of *M. thermophila* and syntrophic co-culture of *M. thermautotrophicus* TM and *T. phaeum* PB were cultivated in medium with pH ranging from 7.0 to 9.0 (Fig. 3B). No significant differences were observed in the methane production rate between pH 7.0 and 8.5 in the syntrophic co-culture, while it was reduced by 70% at pH 9.0.

In contrast, *M. thermophila* PT was severely inhibited by elevated pH with almost no methanogenesis being observed at pH ≥8.0. This could be explained by an increase in the [NH_3_]/[NH_4_^+^] ratio at higher pH conditions because NH_3_ is assumed to more cytotoxic than NH_4_^+^. However, this assumption was ruled out for the following reasons: the methane production rate of *M. thermophila* PT in the 50 mM total ammonia at pH 7 medium (0.65 ± 0.5 d^−1^, Fig. 1) was significantly higher than that in the 5 mM total ammonia at pH 8 medium (0.13 ± 0.8 d^−1^, Fig. 3). The calculated [NH_3_] in these two culture conditions were equal: *i.e.* both [NH_3_]_pH7_ in 50 mM total ammonia and [NH_3_]_pH8_ in 5 mM total ammonia were equal to 0.26 mM. Taken together, these results suggested that the inability of *M. thermophila* PT to grow under high pH conditions may have been caused by its failure in maintaining intracellular pH homeostasis, which may partly account for the susceptibility of this strain to ammonia stress.

### Ammonia inhibition of methanogenesis by *M. thermophila* PT resting cells

**Fig. 4:**
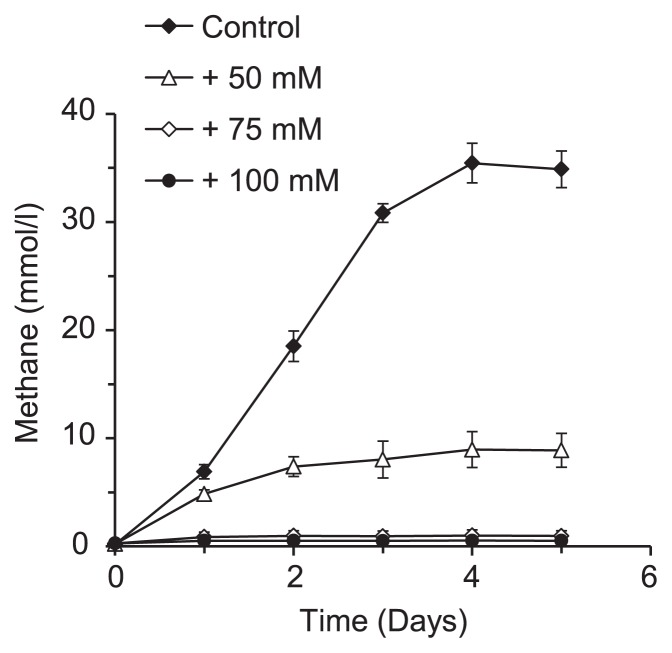
Ammonia inhibition of methanogenesis by resting *M. thermophila* PT cells. Early stationary phase cultures of *M. thermophila* PT grown on 40 mM acetate were flushed with N_2_/CO_2_ (80:20 [v/v]) gas and supplemented with 40 mM acetate and NH_4_Cl, followed by an incubation at 55°C. Data are presented as the means of three independent cultures, and error bars represent standard deviations.

To evaluate the effects of ammonia on the total methanogenic activity of *M. thermophila* PT, the methanogenic activity of the resting cells of *M. thermophila* PT was measured under ammonia stress conditions (Fig. 4). The methanogenic activity of these cells was strongly suppressed by 50 mM NH_4_Cl and was completely inhibited by 75 mM NH_4_Cl. These inhibitory effects were more prominent on resting cells than on cells under growing conditions (Fig. 1). These results suggested that high ammonia concentrations may have had inhibitory effects on the activities of enzymes that may be specifically involved in methanogenesis in *M. thermophila* PT cells.

### Transcriptomic response of *M. thermophila* PT to ammonia stress

**Fig. 5:**
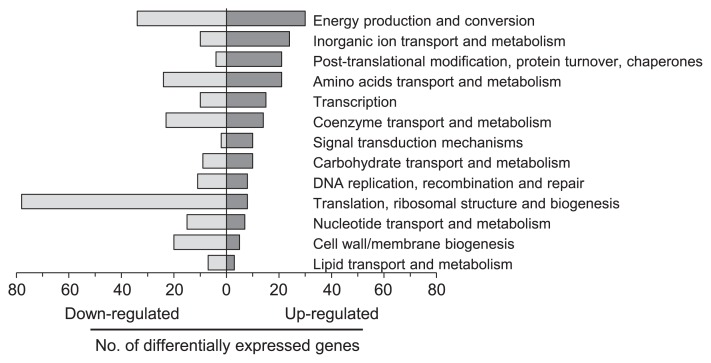
Gene expression patterns of *M. thermophila* PT under ammonia stress. The numbers of significantly up- or down-regulated (Fold > 2 or < −2, *p <* 0.01) genes in each functional category under ammonia-stress conditions are shown.

To gain further insight into the mechanisms of ammonia inhibition, *M. thermophila* PT cells cultivated to the mid-log phase were exposed to 100 mM NH_4_Cl for 1 h and then subjected to transcriptomic microarray analysis. Transcriptome data were compared with those of control cultures without NH_4_Cl supplementation using a two-fold change in gene expression and a *p*-value of 0.01 as cut-off values. The expression of 6 genes for central energy metabolism was examined by qRT-PCR analysis to validate the microarray data (Fig. S2), and the results obtained revealed a strong correlation between these data (*r =* 0.96). Of the 1,671 analyzed genes, 308 (18.4%) and 342 (20.5%) genes were significantly up- or down-regulated, respectively, under ammonia stress conditions. These differentially expressed genes were functionally categorized based on the clusters of orthologous groups of proteins (COG) database (37), as summarized in Fig. 5. The categories in which large numbers of genes were differentially expressed under the ammonia-stress conditions were “translation, ribosomal structure, and biogenesis” (down), “energy production and conversion” (both up and down), “post-translational modification, protein turnover, and chaperones” (up), and “inorganic ion transport and metabolism” (up).

The down-regulation of genes involved in “translation, ribosomal structure, and biogenesis” (particularly genes encoding ribosomal proteins) and “energy production and conversion” (*e.g.*, genes encoding gluconeogenesis pathway enzymes) is a general feature of the microbial transcriptome response to stress-induced growth suppression and is not specific for ammonia stress (15). In the “post-translational modification, protein turnover, and chaperones” category, genes encoding molecular chaperones and antioxidant enzymes were markedly up-regulated in response to ammonia stress (Table S2 and S3), which suggested that the denaturation of intracellular proteins and oxidative stress in *M. thermophila* PT at high ammonia concentrations (for a more detailed discussion, see the Supplemental material). The expression pattern of the genes involved in central energy metabolism, including genes for the methanogenic pathway, categorized in “energy production and conversion” varied from gene to gene (Fig. S3), which was similar to the gene expression profile of *M. thermautotrophicus* ΔH under ammonia stress conditions (15). Although it remains unclear why ammonia stress up- or down-regulated specific energy metabolism enzymes, one plausible explanation is that the inhibition of certain enzymatic activities may have caused the accumulation of intermediate compounds that affect the expression of these genes for up- and down-stream reactions (for a more detailed discussion, see the Supplemental material). In the “inorganic ion transport and metabolism” category, numerous genes of putative ABC-type transporters were up-regulated (Table S4), which is consistent with ammonia stress causing disturbances in intracellular cation balances (33, 40).

## Conclusions

The present study comparatively evaluated the inhibitory effects of ammonia on two different methanogenic acetate degradation pathways, namely aceticlastic and syntrophic pathways, using pure and defined co-cultures. Pure culture or defined co-culture-based studies have yet to be performed. The results presented here demonstrated that the aceticlastic methanogen *M. thermophila* PT was markedly less tolerant to ammonia stress than the syntrophic co-culture of *T. phaeum* PB and *M. thermautotrophicus* TM. The susceptibility of *M. thermophila* PT to ammonia is likely attributed to diverse disruptions under the ammonia stress conditions, including the disruption of intracellular pH homeostasis, inhibition of specific enzymes involved in methanogenesis, protein denaturation, oxidative stress, and intracellular cation imbalances. This study also provided an important insight into the responses of methanogenic communities to ammonia; the presence of two pathways in thermophilic methane reactors confer resiliency and robustness in response to environmental perturbations that are crucial to the key reaction *i.e.* methanogenesis from acetate to circumvent the ultimate collapse of reactor performance.

## Supplementary Information
