# Supplementary material for: Physiological and Transcriptomic Analyses of the Thermophilic, Aceticlastic Methanogen Methanosaeta thermophila Responding to Ammonia Stress
Source: Microbes Environ. 2014 Jun 10;29(2):162–7. doi: 10.1264/jsme2.ME14021 (PMC4103522; doi:10.1264/jsme2.ME14021)
Supplement: Supplementary file 1 [file 29_162_s1.pdf]

Supplemental material

Physiological and Transcriptomic Analyses of the Thermophilic, Aceticlastic Methanogen *Methanosaeta thermophila* Responding to Ammonia Stress

Souichiro Kato[1,2,3]*, Konomi Sasaki[1,4], Kazuya Watanabe[3,5], Isao Yumoto[1] and Yoichi Kamagata[1,2]

[1]*Bioproduction Research Institute, National Institute of Advanced Industrial Science and Technology (AIST), 2-17-2-1 Tsukisamu-Higashi, Toyohira-ku, Sapporo, Hokkaido 062-8517, Japan,* [2]*Division of Applied Bioscience, Graduate School of Agriculture, Hokkaido University, Kita-9 Nishi-9, Kita-ku, Sapporo, Hokkaido 060-8589, Japan,* [3]*Research Center for Advanced Science and Technology, The University of Tokyo, 4-6-1 Komaba, Meguro-ku, Tokyo 153-8904, Japan,* [4]*Hokkaido High-Technology College, 2-12-1 Megumino-kita, Eniwa, Hokkaido 061-1374, Japan,* [5]*School of Life Sciences, Tokyo University of Pharmacy and Life Sciences, 1432-1 Horinouchi, Hachioji, Tokyo 192-0392, Japan*

*For correspondence. E-mail: s.katou@aist.go.jp; Tel.: (+81) 11 857 8968; FAX: (+81) 11 857 8915

Effects of ammonia on the pure cultures of T. phaeum *PB and* M. thermautotrophicus *TM.*

The pure cultures of *T. phaeum* PB and *M. thermautotrophicus* TM were grown on 40 mM pyruvate and 160 kPa of H_2, respectively, with different concentrations of NH_4Cl and their growth rates were determined and compared with that of *M. thermophila* PT (Fig. S1).

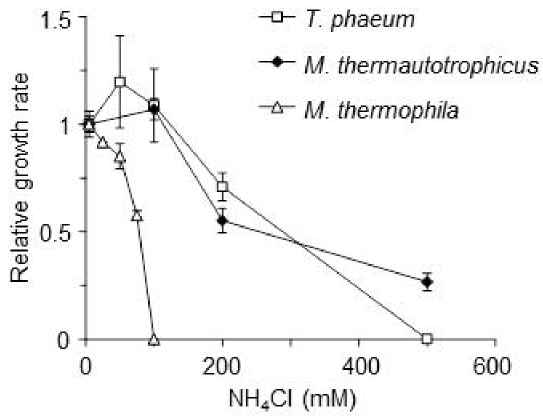

Fig S1. Effects of ammonia on growth rates of pure cultures of the three thermophilic strains. The relative growth rates of *T. phaeum* PB (open squares), *M. thermautotrophicus* TM (filled diamonds), and *M. thermophila* PT (open triangles) are plotted against NH_4Cl concentrations. The growth rates were normalized against those of the control cultures (5 mM NH_4Cl). Data are presented as the means of three independent cultures, and error bars represent standard deviations.

Comparison of qRT-PCR and microarray data.

Six genes for the central energy metabolism of *M. thermophila* PT (listed in the legend of Fig. S2) were selected for qRT-PCR analysis to examine if differential expression levels determined using microarrays were supported by qRT-PCR. Gene expression levels determined by qRT-PCR (copies per ng RNA) after the NH_4Cl treatment were compared with those of control condition, and relative levels were plotted against the microarray data (Fig. S2). The PCR primers used for qRT-PCR are listed in Table S1.

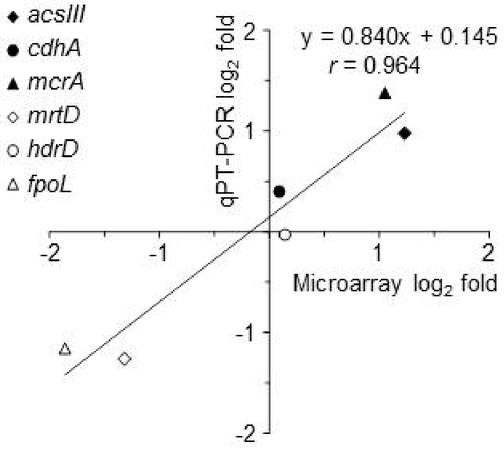

Fig S2. Comparison of qRT-PCR and microarray data. Filled diamonds, acetate CoA-ligase (acsIII, Mthe_1194); filled circles, CO dehydrogenase/acetyl-CoA synthase complex alfa subunit (cdhA, Mthe_0292); filled triangles, methyl-coenzyme M reductase alfa subunit (mcrA, Mthe_0569); open diamonds, tetrahydromethanopterin S-methyltransferase D subunit (mrtD, Mthe_1384); open circles, heterodisulfide reductase D subunit (hdrD, Mthe_0980), open triangles, F_{420}-H_2 dehydrogenase-like complex L subunit (Mthe_1058). The approximation curve and the correlation coefficient (r) are given in the figure.

Table S1. qRT-PCR primers used in this study for amplifying *M. thermophila* PT 16S rRNA and genes for methanogenesis.

Primer name	Target gene	Sequence (5' to 3')
Msaet_387F	16S rRNA	GAT AAG GGG ACC TCG AGT GCT
Msaet_573R	16S rRNA	GGC CGG CTA CAG ACC CT
FpoL_1347F	*fpoL*	CGC TCT GAT TAC CAC AAG CA
FpoL_1465R	*fpoL*	CGA CTG TCT CGT AGA AGC CC
HdrD_1067F	*hdrD*	GGC AGA CAT CAT AGC AAG CA
HdrD_1286R	*hdrD*	TTG CCT GTC ACT GTC TCC AG
MtrD_341F	*mtrD*	TCC GAT CAC CGG ATA CTC TC
MtrD_514R	*mtrD*	GGG CAC TCA GAG ATG GGT TA
CdhA_1567F	*cdhA*	ACT ATC CAA ACG GCA CCA AG
CdhA_1690R	*cdhA*	TGC CCT CTG CAT CAG TGT AG
Acs3_193F	*acsIII*	ACT GGT TCA AGC CGT ACA CC
Acs3_437R	*acsIII*	TTC TTG ACG CCC AGA CTC TT
McrA_1085F	*mcrA*	CGT GGA GAA GTA CGG TGG AT
McrA_1316R	*mcrA*	GAG AGG TAC CAG CCA GCA AG

Up-regulation of chaperone genes under ammonia stress conditions.

In the “post-translational modification, protein turnover and chaperones” category, 5 out of 10 genes encoding molecular chaperones were up-regulated in response to ammonia stress in *M. thermophila* PT (Table S2). The up-regulation of chaperone genes by high ammonia concentrations was also reported for the mesophilic aceticlastic methanogen *Methanosarcina mazei* S-6 (1). In contrast, no chaperone genes were up-regulated in *M. thermautotrophicus* ΔH under ammonia stress conditions (2). Taken together, these findings indicate that the denaturation of intracellular proteins is one of the crucial consequences of ammonia stress in aceticlastic methanogens.

Table S2. Expression of genes encoding chaperone homologues in *M. thermophila* PT under ammonia-stress conditions.

Locus tag	Gene/protein name	Fold change[a]	*p*-value
Mthe_0045	Peptidylprolyl isomerase	1.5	3.8E-3
**Mthe_0053**[b]	**Molecular chaperone (small heat shock protein)**	**3.5**	**2.4E-8**
Mthe_0574	Peptidylprolyl isomerase, FKBP-type	-1.6	3.8E-7
Mthe_0730	Chaperone protein DnaJ	1.0	2.2E-1
**Mthe_0731**	**Chaperone protein DnaK**	**2.0**	**5.1E-5**
Mthe_0732	GrpE protein	-1.7	1.8E-5
**Mthe_0805**	**Heat shock protein Hsp20**	**3.5**	**2.1E-8**
**Mthe_0852**	**Heat shock protein HtpX**	**2.2**	**4.1E-7**
Mthe_1547	Peptidylprolyl isomerase, FKBP-type	1.8	1.7E-4
**Mthe_1631**	**Heat shock protein Hsp20**	**6.4**	**7.3E-6**

a. Fold change in negative values indicate down-regulation under the ammonia stress conditions.

b. Bold letters indicate significantly upregulated genes (Fold > 2, $p < 0.01$).

Up-regulation of genes for antioxidant enzymes under ammonia stress conditions.

In the "post-translational modification, protein turnover and chaperones" category, 10 out of 16 genes, which encode homologues of antioxidant enzymes were up-regulated in *M. thermophila* PT in response to ammonia stress (Table S3). The up-regulation of antioxidant enzyme genes was also reported in ammonia-stressed cells of *M. thermautotrophicus* ΔH (2). Considering that the depletion of methanogenesis substrates, such as H_2, also induces the overexpression of antioxidant enzyme genes in *M. thermautotrophicus* ΔH (2), the suppression of methanogenesis induced by ammonia stress and concomitant undersupply of reducing equivalents required for the reduction of diverse oxidative agents may cause oxidative stress for methanogens.

Table S3. Expression of genes encoding antioxidant enzymes in *M. thermophila* PT under ammonia-stress conditions.

Locus tag	Gene/Protein name	Fold change[a]	*p*-value
**Mthe_0117[b]**	**Rubredoxin-type Fe(Cys)4 protein**	**6.1**	**5.4E-8**
Mthe_0245	Flavoprotein	1.1	2.3E-3
Mthe_0493	Alkylhydroperoxidase like protein, AhpD family	1.0	3.1E-1
**Mthe_0685**	**Superoxide dismutase**	**2.7**	**1.1E-7**
**Mthe_0707**	**Glutaredoxin**	**2.0**	**2.7E-6**
**Mthe_0711**	**Thioredoxin**	**2.0**	**1.3E-5**
**Mthe_0743**	**Rubrerythrin**	**2.2**	**1.1E-8**
Mthe_0782	Alkylhydroperoxidase like protein, AhpD family	-1.6	2.7E-3
**Mthe_0907**	**Rubredoxin-type Fe(Cys)4 protein**	**2.7**	**5.7E-7**
**Mthe_0908**	**Glutaredoxin**	**2.3**	**4.4E-7**
Mthe_1133	Thioredoxin	-1.0	2.7E-1
Mthe_1150	Thioredoxin-disulfide reductase	1.8	1.5E-9
Mthe_1166	Flavoprotein	1.5	4.1E-3
**Mthe_1245**	**Rubredoxin-type Fe(Cys)4 protein**	**3.9**	**2.1E-6**
**Mthe_1416**	**Peroxidase**	**3.9**	**6.6E-9**
**Mthe_1697**	**Thioredoxin-like protein**	**2.6**	**4.1E-8**

a. Fold change in negative values indicate down-regulation under the ammonia stress conditions.

b. Bold letters indicate significantly upregulated genes (Fold > 2, $p < 0.01$).

The expression of genes for the central energy metabolism under ammonia stress conditions.

The expression pattern of the genes involved in the central energy metabolism varied from gene to gene (Fig. S3). For example, the genes encoding acetate-CoA ligase (Acs) and methyl-CoM reductase (Mcr) were significantly up-regulated in cells exposed to ammonia, whereas the genes for F_{420}-H_2 dehydrogenase-like complex (Fpo), ATP synthase (Atp), and two subunits of tetrahydromethanopterin S-methyltransferase (Mtr) were significantly down-regulated. A similar phenomenon was reported for the transcriptome analysis of *M. thermautotrophicus* ΔH: genes encoding a number of methanogenic enzymes, including Mcr, were up-regulated in response to ammonia stress, while the genes for Atp were down-regulated (2). One plausible explanation is that the inhibition of certain enzymatic activities causes the accumulation of intermediate compounds that affect the gene expression of various metabolic enzymes. The inhibition of certain methanogenic enzyme(s) (as suggested by the non-growing culture experiments) causes accumulation of the intermediates (the substrate[s] for the inhibited enzyme[s]) of the methanogenic pathway. Also it is conceivable that accumulation of such intermediate compounds cause up- (or down-)regulation of genes for the down- (or up-)stream reactions. This assumption requires further physiological and molecular biological experiments, for example, (partial) purification of methanogenic enzymes and measurements of the enzymatic activities under various concentrations of ammonia, to confirm it.

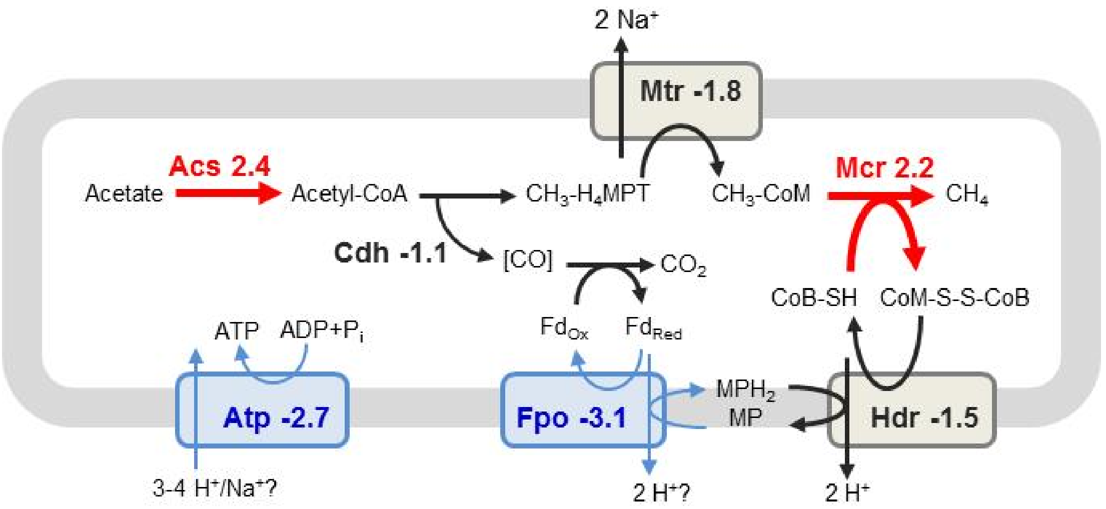

Fig. S3. Schematic image of central energy metabolism of *M. thermophila* PT and gene expression profiles of the respective enzymes. Enzyme abbreviations and fold changes in expression (ammonia stress vs. control) are shown in bold letters. Red and blue letters indicate significantly up- or down-regulated genes (Fold > 2 or < -2, $p < 0.01$), respectively, under ammonia-stress conditions. Acs, acetate-CoA ligase; Cdh, CO dehydrogenase/acetyl-CoA synthase; Mtr, tetrahydromethanopterin S-methyltransferase; Mcr, methyl-CoM reductase; Hdr, heterodisulfide reductase; Fpo, $F_{420}H_2$ dehydrogenase-like complex; Atp, ATP synthase; H_4MPT, tetrahydromethanopterin; P_i, inorganic phosphate; Fd_{Ox} and Fd_{Red}, oxidized and reduced ferredoxin, respectively; and MP and MPH_2, oxidized and reduced methanophenazine, respectively.

Up-regulation of genes for inorganic ion transport under ammonia stress conditions.

Ammonia stress induced the expression of numerous genes in the “inorganic ion transport and metabolism” category, with 25 out of a total of 96 genes being significantly up-regulated in *M. thermophila* PT (Table S4). Among the 25 up-regulated genes, 18 genes were annotated as periplasmic or membrane components of putative ABC-type transporters, although the substrate specificities of these components are largely unknown. These findings are consistent with the speculation that ammonia stress causes disturbance in intracellular cation balances (3, 4).

Table S4. *M. thermophila* PT genes of the "inorganic ion transport and metabolism" category that were significantly up-regulated under ammonia-stress conditions.

Locus tag	Gene/Protein name	Fold change	*p*-value
Mthe_0084	ammonium transporter	2.1	1.1E-4
Mthe_0118	Ferritin, Dps family protein	6.0	5.0E-8
Mthe_0296	hypothetical protein	3.6	1.2E-5
Mthe_0342	ABC-type transporter, periplasmic component	2.1	3.4E-4
Mthe_0344	ABC-type transporter, periplasmic component	2.5	6.5E-6
Mthe_0442	ABC-type transporter, periplasmic component	2.8	7.6E-7
Mthe_0443	ABC-type transporter, periplasmic component	2.8	3.3E-6
Mthe_0444	ABC-type transporter, periplasmic component	8.9	4.1E-8
Mthe_0448	ABC-type transporter, periplasmic component	4.8	6.7E-7
Mthe_0528	ABC-type transporter, periplasmic component	2.1	9.9E-5
Mthe_0685	superoxide dismutase	2.7	1.1E-7
Mthe_0689	rhodanese-related sulfurtransferase	10.3	3.6E-6
Mthe_0858	ABC-type transporter, permease components	2.8	5.7E-5
Mthe_0859	ABC-type transporter, permease components	4.2	1.5E-7
Mthe_0905	heavy metal translocating P-type ATPase	2.1	1.4E-6
Mthe_0972	ABC-type transporter, periplasmic component	3.9	6.8E-6
Mthe_1009	ATPase, P-type (transporting)	4.2	2.2E-7
Mthe_1042	ABC-type transporter, periplasmic component	4.3	1.3E-6
Mthe_1043	adenylylsulfate kinase	2.6	1.4E-8
Mthe_1185	ABC-type transporter, periplasmic component	2.4	1.2E-6
Mthe_1206	Carbohydrate-binding and sugar hydrolysis	2.4	6.1E-5
Mthe_1269	TrkA-N domain protein	2.7	6.4E-6
Mthe_1347	citrate transporter	2.1	1.1E-4
Mthe_1480	phosphate binding protein	23.7	2.9E-9
Mthe_1626	periplasmic copper-binding protein	3.1	1.6E-5

REFERENCES FOR SUPPLEMENTAL

1. Lange, M., A. J. L. Macario, B. K. Ahring, and E. Conway de Macario. 1997. Increased transcripts of the *dnaK* locus genes in *Methanosarcina mazei* S-6 exposed to supraoptimal concentrations of ammonia. FEMS Microbiol. Lett. 152:379-384.

2. Kato, S., T. Kosaka, and K. Watanabe. 2008. Comparative transcriptome analysis of responses of *Methanothermobacter thermautotrophicus* to different environmental stimuli. Environ. Microbiol. 10:893-905.

3. Sprott, G. D., K. M. Shaw, and K. F. Jarrell. 1984. Ammonia/potassium exchange in methanogenic bacteria. J. Biol. Chem. 259:12602-12608.

4. Wittmann, C., A. P. Zeng, and W. D. Deckwer. 1995. Growth inhibition by ammonia and use of a pH-controlled feeding strategy for the effective cultivation of *Mycobacterium chlorophenolicum*. Appl. Microbiol. Biotechnol. 44:519-525.
